# Effectiveness of an 8-week overground walking with paretic lower limb loading on spatiotemporal gait parameters and motor function among chronic stroke survivors: a protocol for randomised controlled trial

**DOI:** 10.1186/s13063-022-07057-3

**Published:** 2023-02-20

**Authors:** Abdulhamid U. Maje, Aminu A. Ibrahim

**Affiliations:** 1 Department of Physiotherapy, Muhammadu Abdullahi Wase Teaching Hospital, Hospitals Management Board, P.M.B 3160, Kano, Kano State Nigeria; 2 Department of Physiotherapy, Faculty of Allied Health Sciences, College of Health Sciences, Bayero University, P.M.B 3011, Kano, Kano State Nigeria; 3 Department of Physiotherapy, School of Basic Medical Sciences, Skyline University Nigeria, Kano, Kano State Nigeria

**Keywords:** Gait symmetry, Lower limb loading, Overground walking, Randomised controlled trial, Spatiotemporal gait parameters, Stroke

## Abstract

**Background:**

Post-stroke gait deviations contribute to significant functional disability, impaired walking ability and poor quality of life. Prior studies suggest that gait training with paretic lower limb loading may improve gait parameters and walking ability in post-stroke. However, most gait training methods used in these studies are not readily available, and studies using cheaper methods are limited.

**Objective:**

The purpose of this study is to describe a protocol for a randomised controlled trial on the effectiveness of an 8-week overground walking with paretic lower limb loading on spatiotemporal gait parameters and motor function among chronic stroke survivors.

**Methods:**

This is a two-center, single-blind, two-arm parallel randomised controlled trial. Forty-eight stroke survivors with mild to moderate disability will be recruited from two tertiary facilities and randomly assigned into two intervention arms; overground walking with paretic lower limb loading or overground walking without paretic lower limb loading in a 1:1 ratio. All interventions will be administered thrice weekly for 8 weeks. Primary outcomes will be step length and gait speed whereas the secondary outcomes will include step length symmetry ratio, stride length, stride length symmetry ratio, stride width, cadence and motor function. All outcomes will be assessed at baseline, 4, 8 and 20 weeks after the start of intervention.

**Discussion:**

This will be the first randomised controlled trial to report the effects of overground walking with paretic lower limb loading on spatiotemporal gait parameters and motor function among chronic stroke survivors from low-resource setting.

**Trial registration:**

ClinicalTrials.gov NCT05097391. Registered on 27 October 2021.

## Background

Stroke remains the second leading cause of death and disability internationally and imposes a considerable personal and societal burden [1]. The health and economic costs associated with stroke are huge and alarming [2, 3]; thus, it poses a major public health problem and warrants development and implementation of optimal primary prevention and rehabilitation strategies to reduce the burden of the disease and its impact [1, 4].

The global incidence and prevalence of stroke have been estimated to be around 11.9 and 104.2 million, respectively [1] and expected to increase in the coming years, especially in low- and middle-income countries [5]. This is more problematic particularly in Sub-Saharan African countries due to limited resources and poor education on stroke risk factors and its warning signs besides the growing and ageing population [4, 6]. The crude prevalence and incidence rates of stroke in Nigeria have been reported to be 1460 per 100,000 and 25 per 100,000 person-years, respectively [7]. Moreover, stroke in Nigeria accounts for approximately 78% of neurological admissions [8], with mortality rates ranging between 21 and 45% [9–13]. The rising prevalence of stroke in Nigeria means the burden may continue to exacerbate pressure on the affected individuals, their families, health and economic systems [14, 15].

Stroke survivors typically exhibit varying neurological deficits and impairments including sensory, cognitive, perceptual, emotional, speech and motor abnormalities [16, 17]. The most important motor deficit post-stroke is hemiparesis contralateral to the cerebrovascular event [17]. This deficit reduces the capacity of the affected lower limb to maintain normal functions such as balance and initiation and control of movements, hence resulting in abnormal gait pattern (i.e. hemiplegic gait) [18, 19].

Post-stroke abnormal gait patterns contribute to significant functional disability, impaired walking ability and poor quality of life [20]. Gait asymmetry implies an imbalance in functional activities between two sides of the body or lower limb [21]. Post-stroke hemiparetic gait is caused by motor impairments such as altered muscle tone, lack of selective motor control, abnormal reflex, poor balance and weakness [22]. Asymmetrical spatial and temporal gait characteristics are common deviations observed in post-stroke hemiplegic gait. It has been documented that about 44–62% and 48–82% of stroke survivors exhibit asymmetry in spatial and temporal gait parameters, respectively [23, 24].

Observational studies have shown that post-stroke hemiparetic gait relative to the normal is significantly characterised by short step length and stride length, reduced cadence, low gait speed, increased step width, double support phases and asymmetric single limb loading [25–28]. After stroke, the paretic limb exhibits a prolonged period of swing and a reduced period of stance; as a result, decreased swing time and increased stance time are seen on the non-paretic limb [29]. These changes are believed to result from decreased walking speed as well as disturbances in other gait parameters between the paretic and non-paretic limbs which may limit gains from rehabilitation [30].

Gait asymmetry does not only affect gait quality in stroke but may also increase energy expenditure [31] and risk of falls [32] and causes loss of bone density of the hemiparetic lower limb [33]. Restoring gait symmetry is, therefore, a major goal for rehabilitation to enhance walking ability and safety, activities of daily living and quality of life among stroke survivors [34].

Despite rehabilitation efforts to improve gait in individuals with stroke, some patients still are unable to walk functionally [35–37]. It appears that many stroke survivors usually do not have significant improvement in gait function [38], and this is often due to inefficient weight application (limb loading) on the affected side causing non-linear limb load transfer [39]. Thus, it is conceivable that adding weight to the paretic limb may correct this imbalance and improve gait performance via an interlimb neural coupling mechanism [40]. Indeed, previous studies on healthy subjects indicate that the addition of small weights to limbs can enhance muscle activation [41, 42], balance ability [43] and walking factors such as distance, velocity and cadence [44].

A previous pretest-posttest pilot study conducted among individuals with chronic stroke found that the addition of a sandbag equivalent to 5% of body weight to ankle of paretic lower limb during weight-bearing treadmill training improved gait velocity, ability to climb stairs and increased ratio of swing phase of both paretic and non-paretic lower limb [45]. In a non-controlled study on chronic stroke patients, the addition of preferred cuff weight (0.7 kg or 1.1 kg) to ankle of paretic lower limb during aquatic treadmill walking decreased unwanted limb floatation and increased stance phase stability [46]. Similarly, in a recent pilot randomised controlled trial (RCT) to determine the effects of aquatic treadmill gait training with water-jet resistance versus underwater treadmill gait training with ankle weights in chronic stroke patients, both training methods improved static and dynamic balance and gait parameters including gait velocity, cadence, step length, stride length and swing phase with water-jet resistance training being more superior [47]. However, underwater treadmill may not be readily available and affordable in low-resource settings. Hence, there is need to devise low-cost and effective methods for gait retraining for stroke survivors in such contexts. It is worth noting that studies in the way of RCTs on the effect of overground walking with paretic lower limb loading in chronic stroke survivors are limited. Compared to underwater or land-based treadmill walk training, overground walk training is easy to perform and relatively cheaper and affordable.

The primary aim of this study is to determine the effectiveness of an 8-week overground walking with paretic lower limb loading compared with overground walking without paretic lower limb loading on step length and gait speed among chronic stroke survivors. The secondary aims were to compare the effects of these interventions on step length symmetry ratio, stride length, stride length symmetry ratio, stride width, cadence and motor function. We hypothesised that overground walking with paretic lower limb loading will have superior effects  on step length and gait speed compared with overground walking without paretic lower limb loading.

## Methods

### Ethical considerations

This study has been approved by the Health and Research Ethics Committee, Ministry of Health, Kano State, Nigeria (ref: NHREC/17/03/2018). The purpose and procedure of the study will be explained to all participants, and their written consent will be obtained.

### Study design

**Fig. 1:**
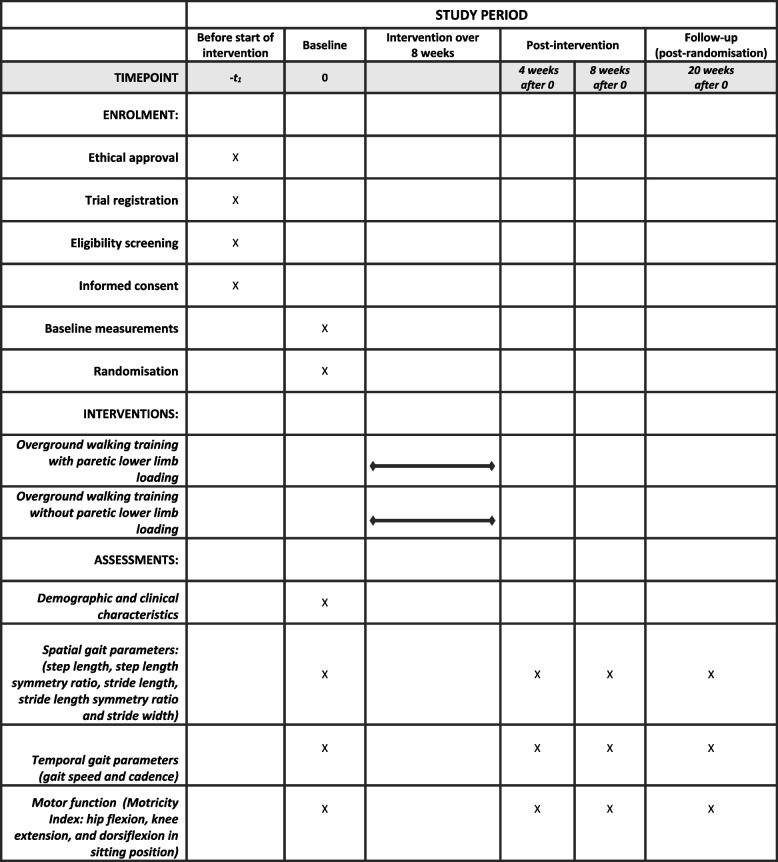
Timetable for enrolment, interventions and assessments

This study will be a single-blind, parallel group, two-arm, RCT with 1:1 allocation ratio. The time schedule of enrolment, interventions and assessments of participants are shown in Fig. 1. The study protocol is reported in line with the Standard Protocol Items: Recommendations for Interventional Trials (SPIRIT) 2013 Checklist (See Appendix I).

### Study setting

The study will be conducted in the physiotherapy outpatient departments of Murtala Muhammad Specialist Hospital (MMSH) and Muhammadu Abdullahi Wase Teaching Hospital (MAWTH), Kano State, Northwestern, Nigeria.

### Training of research assistants

Six licenced physiotherapists (including the primary researcher) with at least five years of working experience in stroke rehabilitation will be engaged in the study. Three therapists including the primary researcher (first author) will be responsible for all aspects of the local organisation including identification, scheduling and screening of potential participants, as well as obtaining written informed consent. The primary researcher with another therapist will administer intervention to the participants. Another two therapists (not involved in screening or administration of intervention) will perform baseline and follow-up assessments. All the research assistants will receive a one-day training session, facilitated by the primary researcher, on the study procedures prior to the beginning of the study. They will be assessed to ensure conversancy and familiarity with the study procedures.

### Participants’ recruitment

The study participants will be drawn from a population of patients with stroke referred for outpatient physiotherapy at MMSH and MAWTH in Kano State. They will be given all the necessary information about the procedures and potential benefits of the study. Written informed consent will be obtained from all the participants after assessment for eligibility. To be eligible for the study, patients must meet the following criteria: (1) unilateral ischemic or hemorrhagic stroke occurring at least 3 months before enrolment; (2) male or female between the age of 20 and 70 years; (3) Having Modified Rankin Scale (MRS) scores of 1, 2 or 3 and also able to walk at least 10 m independently without assistive device; and (4) ability to follow verbal instructions. Patients with one or more of the following criteria will be excluded from the study: (1) cognitive impairments; (2) visual impairments; (3) musculoskeletal disorders that may affect gait such as arthritis; (4) other neurological disorders such as Parkinson's disease, multiple sclerosis; (5) cardio-respiratory conditions that may limit participation such as atelectasis; and (6) concurrent participation in other interventional clinical trials.

Demographic and clinical characteristics such as age, gender, marital status, education level, occupational level, duration of stroke, laterality, leg dominance, blood pressure, weight, height, body mass index (BMI), side of affectation and MRS scores will be obtained using a prepared study proforma. Assessment of baseline outcome variables will be then conducted prior to randomisation. A specific ID will be given to all participants for recognition.

### Randomisation and blinding

**Fig. 2:**
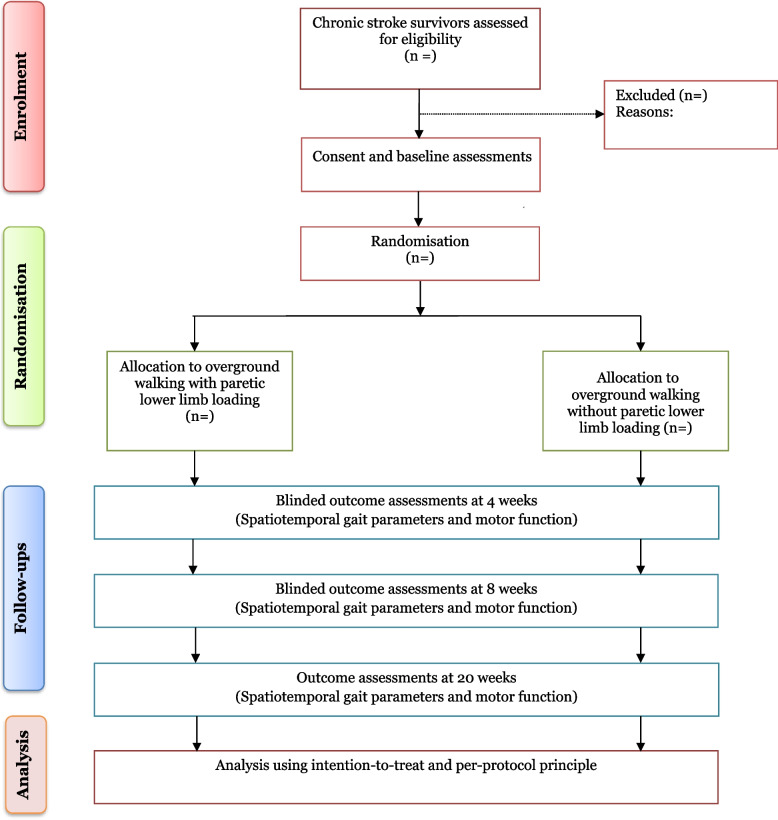
Flowchart showing flow of participants through the study

Upon completion of all the baseline assessments, consenting participants will be stratified by MRS scores (i.e. 1, 2 and 3) and randomised into two groups; overground walking with paretic lower limb loading or overground walking without paretic lower limb loading in a 1:1 ratio (Fig. 2) by a third party not involved in other aspects of the study. Allocation of participants will be concealed using consecutive numbered, sealed and opaque envelopes prepared using a web-based randomisation tool (available at https://www.sealedenvelope.com) with permuted block size of 6. All outcome assessors will be blinded to participants’ group allocation. However, it will be impossible to blind physiotherapists and participants considering the nature of the study interventions.

### Study interventions

#### Overground walking with paretic lower limb loading group

Participants assigned to this group will first receive 5 min warm-up, followed by 15 min conventional exercise, 15–25 min overground walking with paretic lower limb loading, and lastly 5 min cool-down. The warm-up and cool-down will be the same and consist of passive stretching to hamstrings and calf muscles, and a range of motion exercise to the lower limbs. The conventional exercise consists of active-assisted exercises (AAEs) to the lower limbs on the mat, followed by strength training such as sit-to-stand and squatting exercises. For the AAE, assistance will be provided manually by an external force (therapist’s hand) when the participants’ muscle strength is inadequate to complete the motion. For the strength training, participants will perform the sit-to-stand exercise using a comfortable chair at their own pace which will be later progressed to squatting exercises by holding a parallel bar.

The procedure for the overground walking with lower limb loading is similar to that described by Abba et al. [48] except for the addition of weight to the paretic lower limb and increase in intensity of walking in the current study. The walking will be conducted on a flat surface of 15 × 10 m dimensions in the physiotherapy gymnasium. Participants will undergo to and fro overground walk with a 1.5-kg sandbag attached to the paretic limb. The 1.5-kg sandbag was chosen based on the findings of our pilot study (unpublished data) conducted on 18 chronic stroke survivors. We found this weight to be the most comfortable and tolerable weight for overground walking among stroke patients who presented with mild to moderate disability based on the scores of the MRS. Prior to the overground walking, participants will be asked to sit in a relaxed position on a chair with their feet on the ground. The selected weight will be strapped 2 cm above the ankle of the paretic lower limb with the malleoli being the reference point [43]. The strapping will be done in such a way that it is not loose or too tight to give discomfort to the participants while carrying out the training. Afterwards, the participants will be instructed to stand and then walk to and fro a 10-m path (within 15 × 10 m dimensions) labeled with adhesive tape to guide the walking in a straight line. Each participant will be instructed to walk with standardised instruction “walk at your normal, comfortable pace” [49] for 5 min after which they will be asked to stop and rest for 2 min. The training will be repeated 3 times in the first 2 weeks of the study (15 min), 4 times in weeks 3–4 of the study (20 min), and 5 times in weeks 5–8 of the study (25 min). Weight will be unloaded from the paretic limb after completing the training session. The participants will be monitored throughout as they carry out the training, and those reporting symptoms of exertional intolerance outside the target zone (i.e. 12–14) on Borg’s rate of perceived exertion (RPE) scale [50] would be terminated at any time. Treatment will be delivered individually, thrice weekly for 8 weeks. The duration of the training session is expected to last from 40 min in the first week to 50 min in the 8th week. Details of the duration and progression of the study interventions are provided in Table 1.

**Table 1:** Duration and progression of the study interventions

Week	Frequency per week	Warm-up time (min)	Rest time (min)	Conventional exercise time (min)	Rest time (min)	Overground exercise time (min)	Cool-down time (min)	Total rest time (min)	Total exercise time (min)
1^st^	3	5	2	15	2	15	5	4	40
2^nd^	3	5	2	15	2	15	5	4	40
3^rd^	3	5	2	15	2	20	5	4	45
4^th^	3	5	2	15	2	20	5	4	45
5^th^	3	5	2	15	2	25	5	4	50
6^th^	3	5	2	15	2	25	5	4	50
7^th^	3	5	2	15	2	25	5	4	50
8^th^	3	5	2	15	2	25	5	4	50

#### Overground walking without paretic limb loading group

Participants allocated to this group will undergo the same intervention as described for the overground walking with paretic lower limb loading group except that weight will not be applied to the participants’ paretic limb. Treatment will be delivered individually, thrice weekly for 8 weeks. The duration of the training session is expected to last from 40 min in the first week to 50 min in the 8th week. Details of the duration and progression of the study interventions are provided in Table 1.

### Outcome assessments and follow-ups

The primary outcomes will be step length and gait speed whereas the secondary outcomes will be step length symmetry ratio, stride length, stride length symmetry ratio, stride width, cadence and motor function (Table 2). All outcomes will be assessed at baseline prior to randomisation, 4, 8 and 20 weeks after the start of intervention. All participants will be reminded about thier follow-ups via phone call on regular basis.

**Table 2:** Description of primary and secondary outcomes and time points of assessments

S/N	Outcome measure	Description/administration	Psychometrics	Assessment point
	**Primary outcomes**			
1	Step length	It will be measured as the distance from the heel strike of one foot to the heel strike on the next successive step of the opposite foot, using the midpoint of the heel square as the reference point (distance CD measured in cm) See Fig. 3	The measure is easy and has adequate test–retest reliability (*r* = 0.97) [50] and concurrent validity with strobe-light photography [51]	Baseline 4th week 8th week 20th week
2	Gait speed	It will be measured as the distance covered in meters per time taken in seconds to walk a specified distance on a level surface. In this study, it will be calculated as the ratio of 10 m distance per unit time covered during the gait training	The measure has excellent test–retest reliability (ICC = 0.94–0.99) [49, 52] and concurrent validity (*r* = 0.95) with GAITRite system [53]	Baseline 4th week 8th week 20th week
	**Secondary outcomes**			
3	Step length symmetry ratio	It will be calculated by dividing the step length of the paretic limb to that of the non-paretic limb	The calculation has been recommended in determining gait symmetry [28]	Baseline 4th week 8th week 20th week
4	Stride length	It will be measured by taking the midpoint of the heel square as a reference point for measurement, and then measuring the linear distance from the heel strike of one foot to the heel strike of the next successive step of the same foot (distance AB measured in cm). See Fig. 3	The measure is easy and has excellent test–retest reliability (*r* = 0.925) [50] and concurrent validity (*r* = 0.85) with GAITRite system [53]	Baseline 4th week 8th week 20th week
5	Stride length symmetry ratio	It will be calculated by dividing the stride length of the paretic limb to that of the non-paretic limb		Baseline 4th week 8th week 20th week
6	Stride width	It will be measured as the transverse linear distance between points on 2 successive feet. Heel-to-heel step width will be calculated as the difference between distance G and F, measured from the midpoint of 2 successive heel squares of the opposite feet to the edge of the walking path. See Fig. 3	The measure has adequate test–retest reliability (*r* = 0.782) [50] and concurrent validity with strobe-light photography [51]	Baseline 4th week 8th week 20th week
7	Cadence	It will be measured as the number of steps taken per unit time. The value will be calculated by dividing the total number of steps taken on the paper by the total time needed to walk across the length of the floor (measured in steps per minute)	The measure is easy and has excellent test–retest reliability (*r* = 0.905) [50] and good concurrent validity reliability (*r* = 0.75) with GAITRite system [53]	Baseline 4th week 8th week 20th week
8	Motor function (lower limb)	It will be measured with the use of the Motricity Index by assessing hip flexion, knee extension and dorsiflexion in sitting position. The strength of the lower limb will be rated as 0 (no movement); 9 (palpable contraction in muscle, but no movement); 14 (visible movement, but not full range and not against gravity); 19 (full-range movement against gravity, but not resistance); 25 (full movement against gravity, but weaker than the other side); and 33 (normal power) For the hip flexion test, while sitting on a chair, with the paretic hip bent at 90°, the participants will be asked to move the knee towards the chin and the examiner then monitors the contraction of iliopsoas/rectus femoris (anterior thigh). (14 points will be given if there is less than a full range of passive motion; 19 points will be given if the hip is fully flexed yet it can be easily pushed down). For the knee extension test, participants will be asked to sit on a chair with their foot unsupported and the knee at 90°. The examiner will then monitor the contraction of the quadriceps (14 points will be given for less than 50% of full extension; 19 points will be given for full extension yet it can be easily pushed down). For the ankle dorsiflexion test, it will be tested with the foot relaxed in a plantarflexed position. The participant will be asked to dorsiflex the foot and the examiner monitors the tibialis anterior (14 points will be given if there is less than a full range of dorsiflexion)	It is easy to administer and has high inter-rater reliability (*r* = 0.87) and validity (*r* = 0.81 *vs* Rivermead Motor Assessment) for measuring leg impairment in chronic stroke patients [54]	Baseline 4th week 8th week 20th week

#### Measurement of spatial gait parameters

**Fig. 3:**
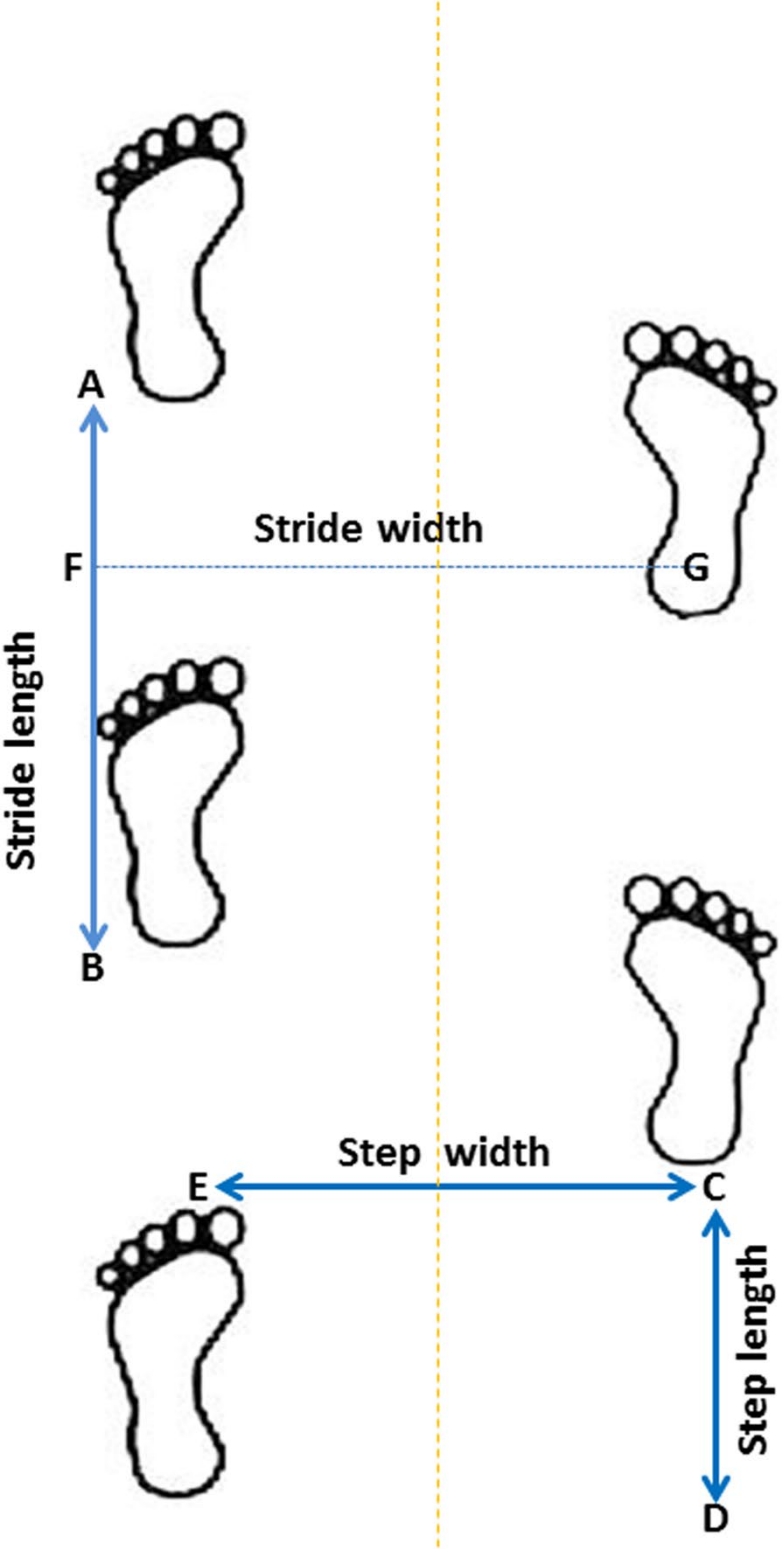
Measurement of stride length (distance AB), step length (distance CD), step width (distance EC) and stride width (FG)

The spatial gait parameters of the participants in both groups will be recorded using the footprint method as described by previous publications [55–57]. Footprints provide a simple and economical method from which step length, stride length, foot angle and stride width can be measured. While sitting on the chair, a pair of flat shoes (slippers) will be given to the participant to wear. Different sizes will be provided to the participants to suit their foot size. All the shoes will be provided with a strap that will be tied to the affected heel to avoid missing steps during the gait training. Ink paste will be applied at the bottom of the participant’s shoes. They will be then asked to stand and walk with the instruction “walk as you normally do” across the 10-m path (as described in the procedure for overground walking training) and footprint sequences from the ground surface will be recorded. The participants will be asked to look directly forward during walking as observing the footprints could alter their gait patterns. At least six footprints will be obtained. The first few steps of the gait footprints will be disregarded because they may not be indicating the stable gait of the participants. Spatial gait parameters will be measured from the footprints using a measuring tape for each of the selected gait components. Each participant will perform three trials of walking and the spatial gait parameters will be recorded in each trial; the average will be then computed. The measurements will be recorded to the nearest centimeters for both the loaded paretic lower limb and non-loaded paretic lower limb during the overground training (Fig. 3). All the parameters will be measured and recorded separately for each leg. The ground floor will be cleaned and mopped to be cleared for the next participant. The spatial gait parameters and symmetry to be measured are fully described in Table 2.

#### Measurement of temporal gait parameters

Similar to the measurement for the spatial gait parameters and symmetry, each participant will perform three trials of walking and the temporal gait parameters (gait speed and cadence) will be recorded in each trial; the average will be then computed. Measurements will be carried out in seconds using a stopwatch each for the loaded paretic lower limb and non-loaded paretic lower limb during the overground training (Table 2).

#### Measurement of motor function

Lower limb motor function of the participants will be assessed using the Motricity Index. Description on how the motor function will be measured is fully provided in Table 2.

#### Adverse events

Adverse events of therapeutic exercise are well known due to their wide application in the healthcare sector. Expected non-serious adverse events related to exercise interventions may include fatigue, falls, muscle soreness or cramps, dizziness and excessive blood pressure responses. Expected serious adverse events include transient ischemic attack (TIA), recurrent stroke, myocardial infarction and sudden death. Based on the findings of our pilot study (unpublished data), no adverse events were reported. However, all participants in this trial will be informed during recruitment of the possibility of experiencing serious and non-serious adverse events. The physiotherapists responsible for treatment will closely monitor the patients during the overground walking training. Adverse events will be reported immediately to the trial management committee which is identical to the authors of this protocol in addition to a physician for prompt action. Adverse events will be also reported to the research ethics committee of Kano State Ministry of Health, Nigeria.

### Sample size calculation

The sample size for this study was estimated using the following parameters: F-test, mixed between-within subjects analysis of variance (ANOVA); alpha (*α*) level of 0.05 (5% chance of drawing a false positive conclusion or committing type I error); power of 0.80 (80% probability of avoiding false negative conclusion; effect size of 0.20 (small effect size was used as no similar RCT could be found); and correlation among repeated measures of 0.5. Based on these assumptions, the total sample size required is 42. However, while accounting for 15% (*n* = 6) dropout rate, a minimum sample size of 48 participants (24 per group) will be finally required. The sample size calculation was performed with G-Power software version 3.1.9.2 for Windows [58]. 

### Statistical analysis

Descriptive statistics of mean, standard deviation, percentage and frequency will be used to summarise the data. Comparison of baseline categorical variables among groups will be conducted using Fisher’s exact test or chi-square test and for continuous variables using independent *t*-test or Mann–Whitney *U* test where applicable. Intention-to-treat principle will be used for the primary statistical analysis by including each participant’s available data in accordance with original allocation and irrespective of the level of attendance. The primary and secondary outcomes will be analysed using linear mixed-effects models (LMMs) by including time (4, 8 and 20 weeks, baseline will be the reference), treatment (overground walking with paretic lower limb loading and overground walking without paretic lower limb loading) and a time × treatment interaction as fixed effects, and a random intercept to account for within-subject correlations. Treatment effect will be defined as the adjusted between-group difference and the associated 95% confidence intervals (CI). We opted to use LMMs because it provides unbiased estimates when data are missing at random [59]. Additionally, per-protocol analysis using the LMMs will be performed as sensitivity analysis by excluding randomised participants who did not received allocated intervention, or who attended fewer than 18 treatment sessions, received proscribed concomitant intervention known to influence gait, and experienced exclusionary medical conditions listed in the study exclusion criteria. All the statistical procedures will be performed using SPSS version 23.0 (IBM Co., Armonk, NY, USA) at a probability level of 0.05.

### Data management

All participants will be recognised only by their ID codes. Data will be stored in secured manner using paper files and in computer hard drive using Microsoft Excel. Participants’ information will be kept strictly confidential and will be accessed only by members of the trial team or ethics committee. Only anonymised data will be made available to other researchers to enable international prospective analyses. All data values will be double-checked by the outcome assessors for errors and missing values before analysis.

### Data monitoring

The study will not have a formal data monitoring committee since the study involves a low-risk intervention. Instead, the authors of this study protocol (identical to trial management committee) will be responsible for monitoring patients’ recruitment, treatment and attrition rates, as well as any other concerns related to the study every 2 weeks.

### Dissemination

The results of this study will be disseminated through conference presentations and publication in peer-reviewed open-access journal irrespective of whether the results are positive, negative or inconclusive.

## Discussion

To our knowledge, no other study in the way of RCT determined the effectiveness of applying additional weight to paretic lower limb during overground walking training on gait ability and motor function compared to overground walking training without paretic lower limb loading among chronic stroke survivors. Individuals with hemiparesis following stroke demonstrate a wide range of spatiotemporal gait abnormalities [25, 26, 60, 61], which in the long run may result in more dependency in basic activities of daily living and consequently reduced quality of life [62]. Improving walking function is therefore the most desired goal of stroke rehabilitation [63] and can be regarded as a yardstick for evaluation [25, 55].

Despite rehabilitation efforts, up to 80% of stroke survivors still experience impaired walking function and further 22% do not regain any walking function [64]. Previous studies [46, 47] found that the addition of weight to paretic lower limb during aquatic walking positively influences spatiotemporal gait parameters of chronic stroke survivors, however, these studies may not be generalised because buoyancy may have influenced some gait outcomes. Another limitation is that they suffered a small sample size with one of them being a non-controlled trial [46]. Moreover, underwater treadmill walking was used for gait training, which may not be readily available and affordable in low-resource settings like Nigerian clinics. This ultimately poses a major challenge, particularly in rural areas where the majority of stroke patients reside [48]. This trial will be therefore conducted to determine the effectiveness of an 8-week, low-cost overground walking with paretic lower limb loading among chronic stroke survivors. Paretic limb loading during overground walking approach could be safe and more accessible as well as affordable compared with aquatic treadmill training.

The present study may further support the evidence of the use of weight loading in gait rehabilitation of stroke patients which may guide clinical practice. A potential limitation of this study is that overground walking on the land-based surface, particularly among older patients and those with excess weight, may increase burden on weight-bearing joints and the risk of falls as compared to underwater treadmill walking. Another limitation is that spatiotemporal gait parameters will not be assessed with gold standard instruments such as three-dimensional (3-D) motion capture system, and electronic walkways, such as GAITRite® [65]. Nevertheless, these instruments are quite expensive to acquire and operate and, as such, the use of technically simple and inexpensive methods like ink footprints may be more feasible for clinical use, especially in resource-constrained settings. Furthermore, because the study will be conducted in two centers, there could be a slight difference in how the therapists perform intervention and assessment.

## Trial status and amendments

The trial registration number is NCT05097391. The protocol version is 1.3. Recruitment of participants is currently ongoing. Recruitment started in November 2021 and is expected to be completed by February 2023. Any justifiable modifications to this protocol will be communicated to Health and Research Ethics Committee, Ministry of Health, Kano State, Nigeria, as well as updated in the ClinicalTrials.gov registry.

## Data Availability

There is no plan to provide public access to the data used for this trial. However, data will be made available by the corresponding author upon request.

## Abbreviations

AAEs: Active-assisted exercises
ANOVA: Analysis of variance
BMI: Body mass index
MAWTH: Muhammadu Abdullahi Wase Teaching Hospital
MMSH: Murtala Muhammad Specialist Hospital
MRS: Modified Rankin Scale
RCT: Randomised controlled trial
SPIRIT: Standard Protocol Items: Recommendations for Interventional Trials
TIA: Transient ischemic attack
